# Unsupervised Bayesian learning for rice panicle segmentation with UAV images

**DOI:** 10.1186/s13007-020-00567-8

**Published:** 2020-02-22

**Authors:** Md Abul Hayat, Jingxian Wu, Yingli Cao

**Affiliations:** 1 Department of Electrical Engineering, University of Arkansas, Fayetteville, 72701 USA; 2 Department of Information and Electrical Engineering, Shenyang Agricultural University, Shenyang, 110041 China

**Keywords:** Rice (*O. sativa*) panicle, UAV, Plant phenotyping, Yield estimation, Image segmentation, Multivariate Gaussian mixture model, Markov chain Monte Carlo

## Abstract

**Background:**

In this paper, an unsupervised Bayesian learning method is proposed to perform rice panicle segmentation with optical images taken by unmanned aerial vehicles (UAV) over paddy fields. Unlike existing supervised learning methods that require a large amount of labeled training data, the unsupervised learning approach detects panicle pixels in UAV images by analyzing statistical properties of pixels in an image without a training phase. Under the Bayesian framework, the distributions of pixel intensities are assumed to follow a multivariate Gaussian mixture model (GMM), with different components in the GMM corresponding to different categories, such as panicle, leaves, or background. The prevalence of each category is characterized by the weights associated with each component in the GMM. The model parameters are iteratively learned by using the Markov chain Monte Carlo (MCMC) method with Gibbs sampling, without the need of labeled training data.

**Results:**

Applying the unsupervised Bayesian learning algorithm on diverse UAV images achieves an average recall, precision and* F*_1_ score of 96.49%, 72.31%, and 82.10%, respectively. These numbers outperform existing supervised learning approaches.

**Conclusions:**

Experimental results demonstrate that the proposed method can accurately identify panicle pixels in UAV images taken under diverse conditions.

## Background

Rice is the most consumed staple food on earth. More than half of the world’s population depend on rice for their daily calories [1]. The yield of a paddy field is directly related to rice panicles, which are the parts of the plant that carry the grains. Fast panicle screening can help rice yield prediction, disease detection, nutrition value assessment, precision irrigation and fertilization, etc [2, 3]. With the rapid development of unmanned aerial vehicle (UAV) and machine learning, there have been growing interests in high throughput rice field phenotyping by using optical images taken by UAVs over paddy fields [4–6].

Image-based rice panicle phenotyping relies on accurate panicle segmentation [7]. One of the main challenges faced by rice panicle segmentation with optical images is the diverse conditions under which the images are taken. There are significant variations among images taken under different conditions, such as water reflections, lighting conditions, weather conditions, cluttering backgrounds, panicle rigidness, rice growth phase, rice strains, UAV altitudes, etc. All these factors will affect the accuracy of panicle identification. This motivates the development of panicle segmentation algorithms that can operate over images taken under a large variety of conditions.

Image-based plant phenomics has gained increasing attentions recently. An automated panicle counting algorithm was developed in [7] by using artificial neural network (ANN). The algorithm was developed by using multi-angle images of rice plants, which was rotated on a turntable to obtain images at multiple angles. In [8], a rice panicle segmentation algorithm, Panicle-SEG, is developed by using deep learning with convolutional neural network (CNN) and superpixel optimization. The Panicle-SEG algorithm is trained with a large number of images, from both pot-grown and field plants, to improve its robustness against the diverse conditions of images. CNN-based deep learning algorithms are also used for rice panicle detection in [9], and for sorghum panicle detection in [10] and [11]. Optical images were also used in [12] for wheat ear detection during the wheat heading stage, and in [13] for studying the flowering dynamics of rice plants. Both [12] and [13] use support vector machine (SVM) for detection. Algorithms mentioned above require a significant amount of labeled training data. More recently, an active learning approach with weak supervision is proposed to reduce the number of labeled training images for panicle detection in cereal crops such as sorghum and wheat [14]. In addition to optical images, hyperspectral images have been widely studied for detecting different plant diseases [15] based on machine learning techniques like principle component analysis (PCA) and chi-square kernel support vector machine (chiSVM) [16, 17].

All above works are based on supervised learning, which requires a substantial number of labeled images for training. To the best of our knowledge, no unsupervised learning method has been developed or applied for rice panicle segmentation. The performance of supervised segmentation algorithm relies heavily on the quality of the training data set. Due to the diverse conditions of rice fields, there are significant variations in the statistical properties of pixels from different images. For example, the illumination and weather condition will have big impacts on the statistical distributions of panicle pixels in different images. Even though the supervised algorithm can be trained by using a large number of images taken under different conditions, it is almost impossible for to capture the large variations among different images by using a single trained model. As a result, for an algorithm trained with one set of images, it might not perform well in other sets of images taken at different conditions. This motivates us to develop an unsupervised learning algorithm that can learn, identify, and adapt to the underlying statistical properties of each individual image, thus works well under all conditions.

The objective of this paper is to develop an unsupervised Bayesian learning algorithm for rice panicle segmentation with UAV images. The algorithm performs panicle detection by identifying the inherent differences in statistical distributions between panicle pixels and non-panicle pixels within the same image, without the need of a training stage. The difference in statistical distributions can then be used to classify the pixels into different categories. The algorithm adopts a probabilistic learning approach that can iteratively calculate the probability of each pixel in an image belonging to different categories, such as panicle, leaves, and background. Such a probabilistic approach can quantify the uncertainty regarding the detection results that is not available in conventional deterministic approaches. Under the Bayesian framework, a multivariate Gaussian mixture model (GMM) is used to represent the pixel intensities in one image, with each component in GMM corresponding to one possible category. With the unsupervised learning approach, the model parameters are directly learned by using unlabeled data from each individual UAV image. Different images will have different model parameters, and this makes the algorithm adaptable to images taken under a wide variety of conditions. Markov chain Monte Carlo (MCMC) [18] with Gibbs sampling [19–21] is employed to learn and update the model parameters. Experimental results demonstrate that the unsupervised Bayesian learning approach can achieve accurate panicle segmentation with UAV images, and it outperforms existing supervised learning approaches. Moreover, this algorithm can also be used in active learning and semi-supervised learning models.

## Results

**Fig. 1:**
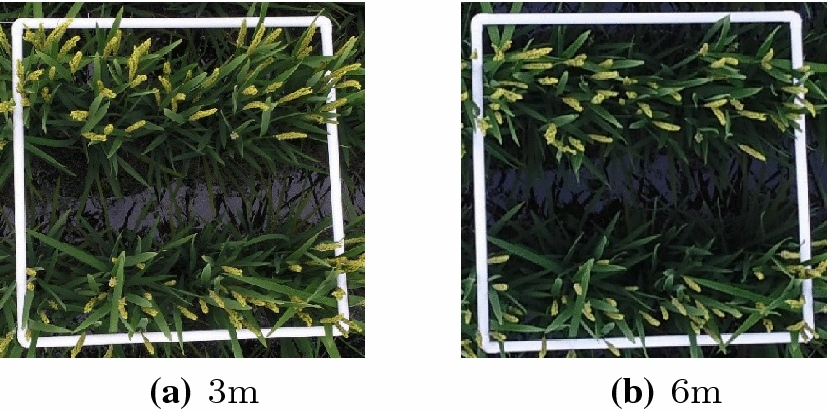
Images of one sampling square taken at different altitudes

The proposed unsupervised Bayesian learning algorithm is applied to the UAV images for panicle segmentation. The UAV images were stored in RGB format, with each pixel represented by a $p=3$ dimension vector corresponding to the colors of red, green and blue, respectively. The value of each color is normalized to the range between 0 and 1. A total of 12 images were processed by the algorithm. Among them, images 1 to 6 were taken at an altitude of 3 m, and images 7 to 12 were taken at an altitude of 6 m. The average spatial resolution (distance between two adjacent ground samples) for 3 m and 6 m images are 0.52 mm and 1.17 mm per pixel, respectively. Figure 1 shows two images of one square segment taken at an altitude of 3m and 6m, respectively. The images were acquired during middle heading stage of rice on August 21, 2017 and September 1, 2018, respectively. The measurements were carried out between 10:00 a.m. and 2:00 p.m. The weather condition on those two days were sunny with a temperature between 21 and 31 °C and class 1–2 south wind, and sunny with a temperature between 19 and 28 °C and class 1–2 northeast wind, respectively. Table 1 shows the detailed information of all 12 images studied in this paper.

**Table 1:** Information of the UAV images

Image	Altitude (m)	Image resolution	% of panicle pixels	Spatial resolution (mm)
1	3	$820 \times 865$	3.67	0.60
2	3	$810 \times 800$	4.74	0.62
3	3	$1050 \times 1075$	7.09	0.47
4	3	$1050 \times 1050$	5.22	0.48
5	3	$1080 \times 1040$	7.36	0.47
6	3	$1120 \times 1070$	6.89	0.46
7	6	$415 \times 410$	5.64	1.21
8	6	$440 \times 415$	8.53	1.17
9	6	$430 \times 430$	3.97	1.16
10	6	$445 \times 440$	7.07	1.13
11	6	$430 \times 430$	7.78	1.16
12	6	$430 \times 420$	5.48	1.18

**Fig. 2:**
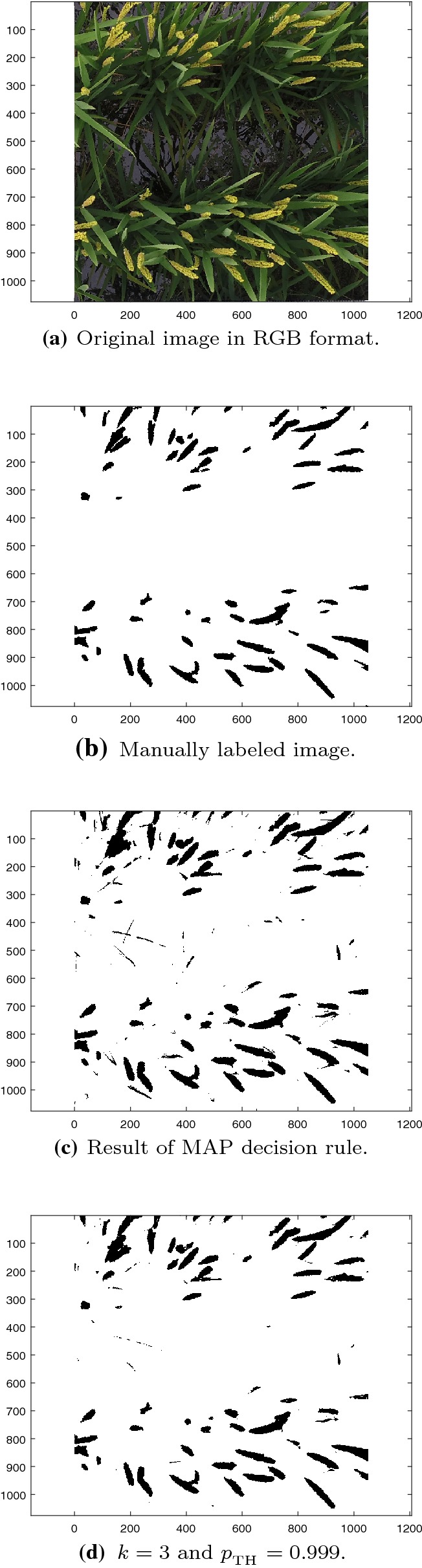
Segmentation results of Image 3

To evaluate the accuracy of the unsupervised Bayesian learning algorithm, the pixels in all UAV images were manually labeled into panicle segments and non-panicle segments, respectively. The manually labeled results are used as a benchmark for evaluation. A pixel-by-pixel comparison is performed between the manually labeled images and automatically segmented images to quantitatively evaluate the results of the proposed algorithm. The percentage of panicle pixels in Table 1 is obtained by using the manually labeled results. As an example, Fig. 2a, b show Image 3 in RGB format and the corresponding manually labeled results, respectively.

In the Bayesian learning algorithm, the initial parameters for the Dirichlet distribution is set as $\alpha _1 = \cdots = \alpha _k = 1$. The prior mean of the mean vector ${\varvec{\mu }}_j$ of the GMM model is set as ${\varvec{\tau }}_1 = \cdots = {\varvec{\tau }}_k = \varvec{0}_p$, where $\varvec{0}_p$ is a length-*p* all-zero vector. The prior precision matrix of the mean vector ${\varvec{\mu }}_j$ is set as $\varvec{\Omega }_j^{(0)} = 10^{-3} \varvec{I}_{p \times p}$. All values of the parameters used in the algorithm are summarized in “Methods” section. All results are based on $T = 150$ iterations in Gibbs sampling, and samples from the first $T_0 = 75$ iterations are discarded before evaluation. The pixels in each image are classified into one out of $k = 3$ categories: panicle, leaves, and dark background.

Let $\mu _{i,j}$ represent the mean of the *j*-th color channel in *i*-th class, where $i \in \{1,2, \ldots , k\}$ and $j \in \{r,g,b\}$. Define $m_i$ as the total mean across all channels for class *i* as

$$\begin{aligned} m_i = \sum _{j \in \{r,g,b\}} \mu _{i,j}. \end{aligned}$$

Based on our experiment results, panicle pixels have the largest total mean across all channels, followed by the leaves and background, respectively. Thus the panicle class can be detected as

$$\begin{aligned} c = {\mathop {{{\,\mathrm{argmax}\,}}}\limits _{i}} \quad m_i. \end{aligned}$$

Since the manually labeled results identify only panicle and non-panicle pixels, the automatically classified pixels belonging to the leaves and background categories are grouped together as non-panicle pixels before comparison.

Figure 2c shows the classification results of Image 3 with the unsupervised Bayesian learning algorithm and the MAP (maximum a posteriori) decision rule as shown in Algorithm 2 in “Methods” section. A visual comparison between Fig. 2b and c indicates that the automatically detected results are strongly correlated with the manually labeled results.

**Fig. 3:**
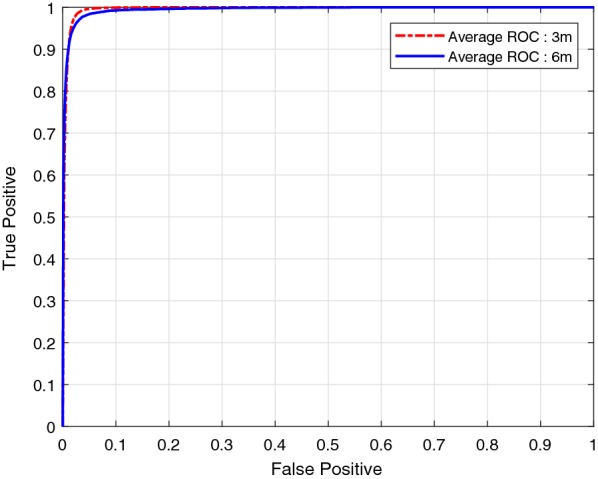
Average ROC curves

To quantitatively evaluate the performance of the unsupervised Bayesian learning algorithm, Fig. 3 shows the average receiver operation characteristic (ROC) curves of the proposed algorithm. Each ROC curve is obtained by averaging over all images at the same altitude. Each point on the ROC curve is obtained by adjusting the threshold of the posterior probability of the panicle category. The tradeoff between the probabilities of true positive (TP) and false positive (FP) can be adjusted by tuning the threshold $p_{_{\text {TH}}}$. In this case the goodness of performance depends on lower FP, thus proper selection of $p_{_{\text {TH}}}$ is important. As example, for Image 3, a TP probability of 0.9788 is achieved with a FP probability of 0.0144 by setting $p_{_{\text {TH}}}= 0.9990$. Based on the ROC results, the algorithm operates equally well for images obtained at both 3m and 6m, with the performance of the 3m images slightly better than that of the 6m images. Averaged over all 12 images, the unsupervised Bayesian learning algorithm can achieve an average recall of 96.49% with average precision of 72.31%, and this is achieved by setting $p_{_{\text {TH}}}= 0.9990$. Figure 2d shows the segmentation result of Image 3 by setting $p_{_{\text {TH}}}= 0.9990$.

**Fig. 4:**
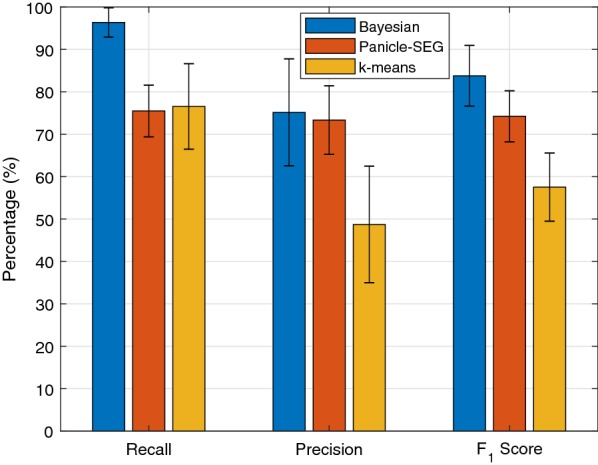
Average performance for 3 m images

**Fig. 5:**
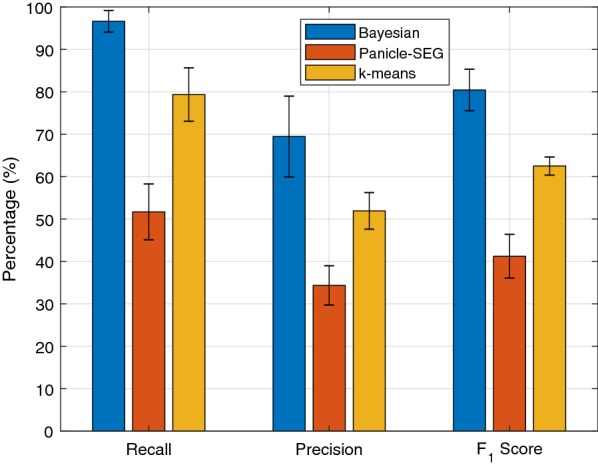
Average performance for 6 m images

The detection results of all 3 m and 6 m images are tabulated in Tables 2 and 3, respectively. The results from *k*-means clustering [22] and Panicle-SEG [8] are also shown in Tables 2 and 3 for comparison. The *k*-means clustering is an unsupervised algorithm aiming to minimize the within-cluster variation after assigning each observation to one of the *k* clusters. In this paper, the within cluster variation is measured by using Euclidean distance, and the algorithm is implemented with the “*k*-means++” algorithm [23], which is the default *k*-means implementation in MATLAB. The Panicle-SEG algorithm is based on a pre-trained model with both in-lab and field measurements of 684 images, including 49 top-view field rice images, 30 overhead-head view field rice images, 302 pot-grown rice side-view images, and 303 pot-grown rice top-view images [8], and the pre-trained model is available online for download [24]. The balancing parameter and optimization coefficient for Panicel-SEG are set as 0.5 and 0.9, respectively, for 3m images, and they are set as 0.5 and 0.8, respectively, for 6m images. In addition, Figs. 4 and 5 compare the performance of the three algorithms by averaging over all images obtained at the same altitude.

**Table 2:** Comparing results of 3 m images

Image	Recall			Precision			$F_1$ score		
	Bayesian	P-SEG	*k*-means	Bayesian	P-SEG	*k*-means	Bayesian	P-SEG	*k*-means
1	0.9735	0.6811	0.8701	0.5925	0.6718	0.2896	0.7367	0.6764	0.4346
2	0.9847	0.7140	0.8341	0.6427	0.6060	0.4341	0.7778	0.6556	0.5710
3	0.9788	0.8580	0.7151	0.8382	0.7314	0.5752	0.9030	0.7897	0.6375
4	0.9915	0.7761	0.8508	0.6929	0.7849	0.3930	0.8158	0.7805	0.5377
5	0.9546	0.7374	0.6188	0.8348	0.7946	0.6544	0.8907	0.7649	0.6361
6	0.8977	0.7621	0.7037	0.9079	0.8123	0.5775	0.9028	0.7864	0.6344

**Table 3:** Comparing results of 6 m images

Image	Recall			Precision			$F_1$ score		
	Bayesian	P-SEG	*k*-means	Bayesian	P-SEG	*k*-means	Bayesian	P-SEG	*k*-means
7	0.9752	0.5583	0.8519	0.6856	0.3284	0.5209	0.8052	0.4136	0.6465
8	0.9166	0.5963	0.7234	0.8839	0.4066	0.5885	0.8999	0.4835	0.6490
9	0.9873	0.4124	0.8838	0.6242	0.2655	0.4612	0.7649	0.3231	0.6061
10	0.9699	0.5048	0.7387	0.6764	0.3505	0.5187	0.7970	0.4137	0.6094
11	0.9816	0.5506	0.7840	0.6456	0.3495	0.5363	0.7789	0.4276	0.6369
12	0.9669	0.4794	0.7797	0.6526	0.3621	0.4907	0.7793	0.4126	0.6023

It is evident that the Bayesian based method consistently outperformed Panicle-SEG and *k*-means algorithm for all the images considered in this paper. For the 3 m images, the proposed algorithm can achieve an average recall of 96.35% with an average precision of 75.15%. These two values are 75.48% and 73.35% for Panicle-SEG, and 76.54% and 48.73% for *k*-means. The corresponding $F_1$ score of the three algorithms are 83.78%, 74.23%, 51.52%, respectively, for all 3m images. Therefore, compared to the Panicle-SEG algorithm, the proposed algorithm can achieve a much higher recall with a similar precision for 3m images, which results in a significantly improved $F_1$ score.

For 6m images, the performance of the Bayesian based method and the *k*-means algorithm remain similar to those from the 3m images. However, the performance of the Panicle-SEG method drops significantly for the 6m results. The recall and precision of the Bayesian based method are 96.23% and 69.47%. These two metrics for the Panicle-SEG algorithm drop to 51.70% and 34.38%, and they are 79.36% and 51.94% for the *k*-means algorithm. Consequently, the $F_1$ scores of the Bayesian based method, Panicle-SEG, and *k*-means are 80.42%, 41.24%, and 62.50%, respectively.

The performance degradation of the Panicle-SEG is partly due to the fact that a lot of the training images are taken at close range with pot plants. On the other hand, the unsupervised learning approach can automatically adjust to different altitudes and achieve similar performances regardless of the altitude differences. This again asserts the versatility and adaptability of the Bayesian based unsupervised learning approach.

## Discussion

The data used in this paper was collected in a sunny and uncloudy day during the middle heading stage of rice. The proposed algorithm relies on the brightness of the pixels, so proper care should be taken to ensure uniform brightness within each image. Failure to maintain this condition can seriously deteriorate the performance. Weather, like other methods [8], is an important factor when the performance is evaluated. As long as panicle pixels and non-panicle pixels maintain different Gaussian distribution this algorithm is going to work quite efficiently irrespective of the height at which the images are captured. In higher altitudes UAV can scan the field with less number of images rendering faster implementation of this algorithm. The results presented in this paper are obtained using just one variate of rice. Results may vary depending on rice variate, rigidness and brightness of rice panicle. All simulations have been done using custom routines in MATLAB. The computer used in the simulation was equipped with 8GB RAM and Intel Core i7-4790 processor. No GPU or parallel computing paradigms have been used. As the number of pixels in 3m images are almost 6 times compared to number of pixels in 6m images, the 6m images are much faster to process. $T_0$ has been chosen to make sure that the samples drawn from following iterations are almost from a stationary distribution. Also, non-informative prior for precision matrix has been used in this paper for faster implementation. Informative priors of precision matrix can also be used.

### Multivariate Gaussian distribution

**Fig. 6:**
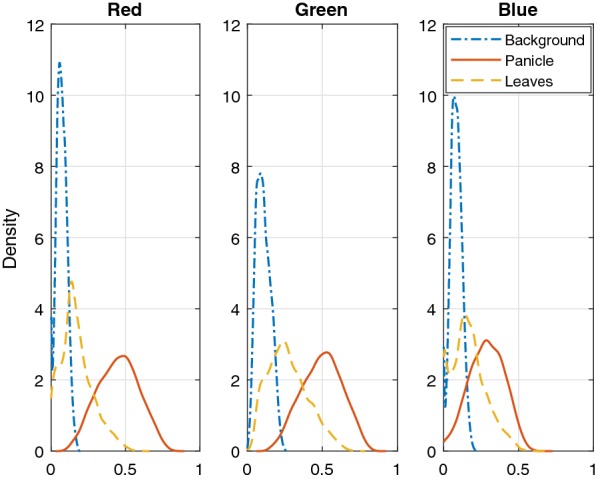
Probability density function of pixels after estimation of classes with $k = 3$ from Image 7

Figure 6 illustrates probability density functions of pixels under three different classes obtained from Image 7. The probability density functions are obtained by using the classification results from the Bayesian based method. As can be seen from the estimated distributions, the pixels in different color channels and under different categories roughly follow Gaussian distributions which justifies the selection of Gaussian distribution in this mixture model. The principle of maximum entropy [25] states that for a given mean and variance, the Gaussian distribution has the maximum entropy among all distributions. Even if the actual distribution of the underlying data is not Gaussian, under the same mean and variance, the Gaussian assumption represents the worst case with the maximum uncertainty. Thus the Gaussian assumption is a good starting point when the prior knowledge of the actual distribution is not known. Due to the above two reasons, the multivariate Gaussian distribution is used to model the pixel distributions under different clusters.

### Number of classes

**Fig. 7:**
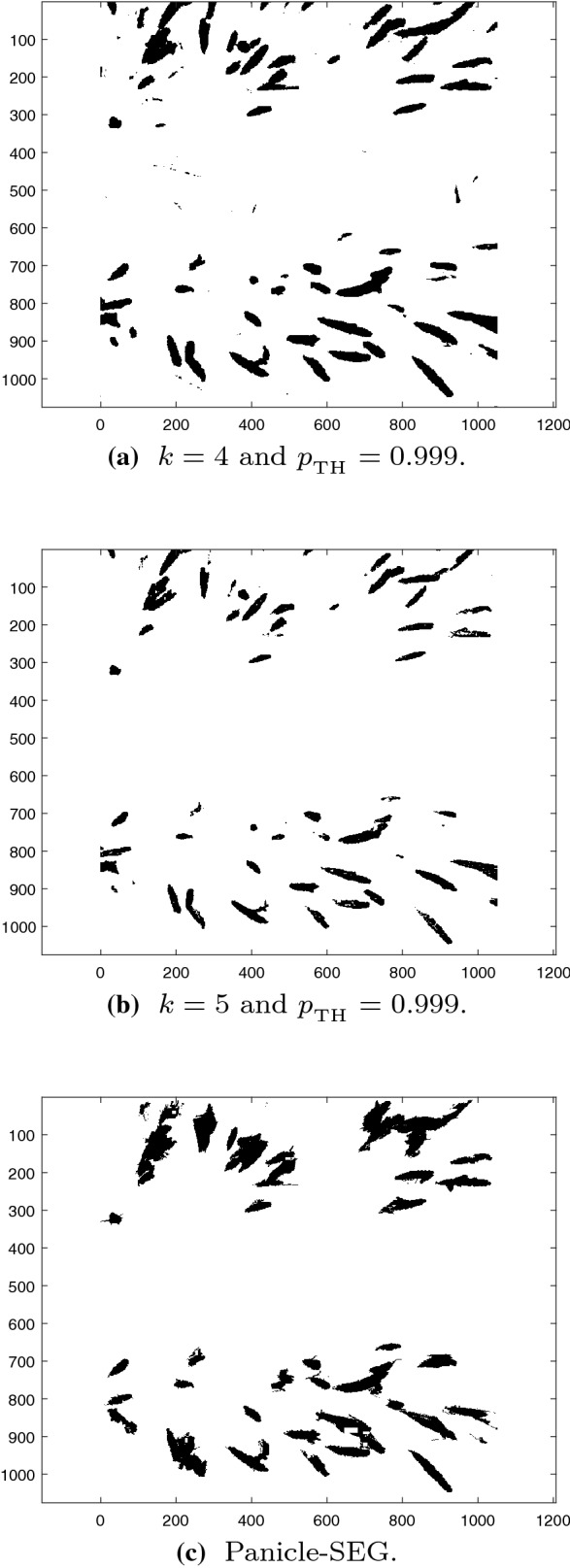
Segmentation results of Image 3 with $k =4$ and 5 and Panicle-SEG

Under different illumination conditions the leaf pixels might correspond to multiple categories due to reflection, diffusion, and shadowing. In that case the number of categories *k* can be increased to capture diverse conditions, and some of the clusters close to each other can be combined later before detection. Details of the method of determining the number of clusters are given in Algorithm 3 in “Methods” section. Since the objective is to identify panicles, all categories other than panicles are grouped together at final output of the segmentation. Segmentation results of Image 3 with $k=4$ and $k = 5$ are in Fig. 7. The recall, precision, and F1 score for Image 3 with $k = 3$, 4, and 5 categories are summarized in Table 4. For $k = 3$ and 4, the results are almost the same, but the performance drops considerably when *k* is increased to 5. Setting *k* too high creates unnecessary categories that will negatively affect the performance. Therefore, the number of categories should depend on the illumination conditions to achieve better classification results.

**Table 4:** Segmentation results of Image 3

*k*	Recall	Precision	F1
3	0.9788	0.8382	0.9030
4	0.9804	0.8016	0.8820
5	0.7326	0.9831	0.8396

### Robustness against anomaly object

**Fig. 8:**
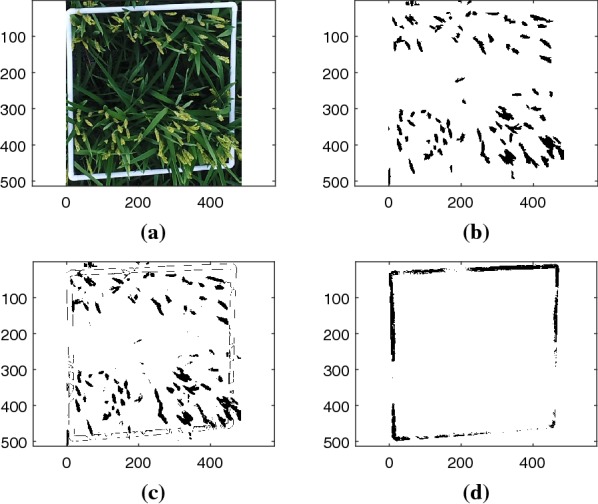
**a** RGB image (6m) with white anomalous rectangle; **b** ground truth of panicle pixels; **c** detected panicle pixel; **d** detected anomaly object

The proposed algorithm is robust against the existence of anomaly objects. In case of an anomaly object, the number of clusters *k* can be increased to account for the distribution of pixels of the anomaly object. Cluster *i* is anomaly if the total mean of a cluster across all channels is greater than a predefined threshold $\epsilon _\text {a}$, as $m_i \ge \epsilon _\text {a}$. Details of anomaly detection is discussed in Algorithm 3 in “Methods” section. In this paper $\epsilon _\text {a}= 0.9$ has been used. Figure 8 shows an image with a white rectangle used to mark the rice field. The image is segmented by using $k = 4$ clusters. After classification, the white rectangle has the highest average mean across channels (0.9239), and the panicle pixels have the second highest average mean across channels (0.4973). Detection results for panicle and anomaly object are shown in Fig. 8c, d, respectively. With the existence of the anomaly object, the recall, precision, and F1 values for panicle pixels with $p_{_{\text {TH}}}= 0.999$ are 0.86, 0.80, and 0.83 respectively.

### Spatial information

The Bayesian based method treats all pixels as independent in the spatial domain whereas the spatial information is utilized by the Panicle-SEG method. The omission of spatial information in the Bayesian based method can sometimes lead to the misclassification of stems as panicles as shown in Figs. 2d and 7a. Such misclassification is not present in the Panicle-SEG as shown in Fig. 7c. However, a larger number of background pixels surrounding the panicles are misclassified as panicles by the Panicle-SEG algorithm, which leads to a relatively high false positive rate in Panicle-SEG. The CNN of Panicle-SEG was trained on patches of $32 \times 32$ pixels thus some panicle pixels remain undetected in rectangular region because of this patch based training. This phenomenon will also increase false negative rate in Panicle-SEG and it gets worse in 6m low resolution images resulting low recall values. As a result, the performance of Panicle-SEG degrades significantly for 6 m images as shown in Table 3.

## Conclusions

The rice panicle segmentation in UAV images with unsupervised Bayesian learning has been studied in this paper. The unsupervised learning approach does not require a training phase, which makes it extremely useful for dealing with images taken under diverse conditions and at different UAV altitudes. Each pixel in the UAV image was modeled by using the multivariate GMM, and the model parameters of different categories were iteratively learned from the UAV data by using MCMC with Gibbs sampling. Experimental results demonstrated that the proposed algorithm can detect panicle pixels in UAV images with very high accuracy, and it outperforms existing supervised learning approach such as panicle-SEG. To the best of the authors knowledge, there does not exist any unsupervised method for panicle segmentation in the literature.

For future works, the results from this paper will be leveraged to estimate the number of rice panicles in a unit area. The results can be used to predict the rice yields for a given field by building new statistical models linking yields with panicle counts in UAV images. In addition, in this paper each pixel is assumed to be independent from neighboring pixels but in practice neighboring pixels are dependent on each other. It is expected that the performance can be further improved by considering spatial dependence among the pixels.

## Methods

### Experiment setup and data collection

**Fig. 9:**
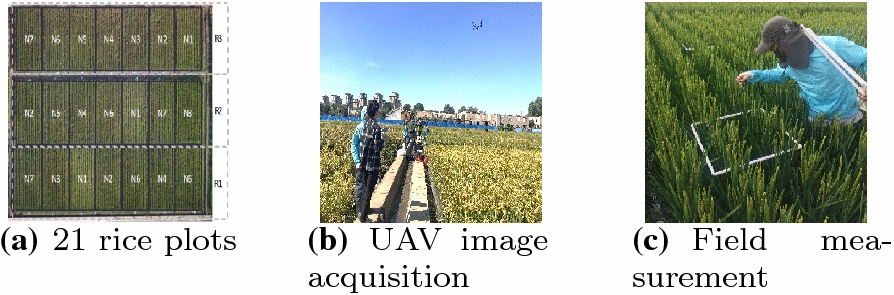
Experiment setup at the Super Rice achievement Transformative Base of SYAU. (N1–N7: nitrogen application levels; R1–R3: three replicates)

The field experiments were conducted in 2017 and 2018 at the Super Rice achievement Transformative Base (SRTB) (E $123^\circ 55^{\prime }85^{\prime \prime }$, N $41^\circ 81^{\prime }63^{\prime \prime }$) of the Shenyang Agricultural University (SYAU) in northeastern China. Shenyang has a temperate semi-humid continental climate, where annual mean temperatures range between 6.2 and 9.7 $^\circ$C and rainfalls range between 600 and 800 mm. Both experiments were performed during middle heading stage of rice using a randomized complete block design with 7 types of nitrogen treatments (N1–N7). The seven nitrogen fertilizers were: null N (0 kg/ha), low N (150 kg/ha), moderate N (240 kg/ha), high N (330 kg/ha), organic fertilizer substitution $10\%$, organic fertilizer substitution $20\%$, and organic fertilizer substitution $30\%$. Each nitrogen treatment has three replicates (R1–R3), which result in a total of 21 plots, as shown in Fig. 9a. Each plot has an area of 30 $\mathrm {m}^2$ (4.2 m $\times$ 7.61 m), separated by dirt paths. The rice cultivar was Shennong 9816.

Images were acquired during middle heading stage of rice on August 21, 2017, and September 2, 2018, respectively, using unmanned aerial vehicles (UAV). The UAV platform was Inspire2 with ZENMUSE X5S camera (15 mm focal length, 20.8MP, 5280 $\times$ 3956 pixels). Images were taken from 3 and 6 m above rice canopy (Fig. 9b). Each image represents a 0.5 m $\times$ 0.5 m white square segment distributed in the middle and edge areas of the plots. Totally 126 images were collected and each image was standard RGB image in unit8 data format with .jpg encoding. The measurements were carried out between 10:00 a.m. and 2:00 p.m. when it was sunny and uncloudy. Field measurement were performed to manually count the number of panicles in each sampling square right after image acquisition (Fig. 9c). Figure 1 shows two images of one square segment taken at an altitude of 3 m and 6 m, respectively.

During our experiment, there was significant downwash effects when the flight altitude is 1 m or less. Under such condition, the downwash effect makes it difficult for the camera to achieve proper focus on the plants, and the correspondingly acquired images are out of clarity. However, there was almost no downwash effects when the altitude is 2 m or higher based on our aerial experiment.

### Problem formulation with Bayesian mixture model

Assume each UAV image contains *n* pixels. The *i*-th pixel in an image can be represented as a *p*-dimension vector as

1 $$\begin{aligned} \varvec{x}_i = [x_{i1}, x_{i2}, \ldots , x_{ip}]^{T}, \quad \text {for} i = 1, \ldots , n. \end{aligned}$$

where $\mathbf{a }^T$ represents matrix transpose. For a regular optical camera, we have $p = 3$, with the three dimensions corresponding the the intensities of red, green, blue of the pixel. The UAV image can thus be represented as the collection of the *n* pixels as $\mathbf{X }= [\mathbf{x }_1, \mathbf{x }_2, \ldots , \mathbf{x }_n]$.

Each pixel can be classified into one of *k* categories, such as panicles, leaves, dirt, water, etc. Define a sequence of independent latent variable $z_i \in \{1,2,\ldots ,k\}$, for $i = 1, 2, \ldots , n$. The latent variables are used to indicate the classification result, that is, $z_i = j$ means that the *i*-th pixel belongs to the *j*-th category, for $j = 1, \ldots , k$. Define $\varvec{z} = [z_1, z_2, \ldots , z_n]^T$. It is assumed that the latent variables follow a multinomial distribution, with the probability mass function (PMF) of $z_i$ represented as

2 $$\begin{aligned} \pi (z_i = j) = q_j, \end{aligned}$$

where $q_j$ is the prior probability of *i*-th pixel belonging to the *j*-th category.

In Bayesian inference, the prior probability vector $\varvec{q} = [q_1, q_2, \ldots , q_k]^T \in \mathcal {L}^{k \times 1}$ with $\mathcal {L} \in [0, 1]$ is unknown and is usually assumed to be a random vector that follows the Dirichlet distribution, i.e.,

3 $$\begin{aligned} \pi (\varvec{q}) = \text {Dir}(\varvec{\alpha }), \end{aligned}$$

where $\varvec{\alpha } = [\alpha _1, \alpha _2, \ldots , \alpha _k]^T \in \mathbb {R}^{k \times 1}$ represents the parameter of the Dirichlet distribution.

The objective of the unsupervised classifier is to identify the value of $z_i$, for $i = 1, \ldots , n$, based on the UAV image data $\mathbf{X }$. The optimum classifier that can minimize the classification error is the maximum a posterior probability (MAP) classifier, which maximizes the posterior probability of $z_i$ as

4 $$\begin{aligned} \hat{z_i} = {\mathop {{{\,\mathrm{argmax}\,}}}\limits _{j \in \{1,\ldots ,k\}}} \Pr (z_i = j|\mathbf{X }), \end{aligned}$$

where $\hat{z_i}$ is the classification results, and $\Pr (z_i|\mathbf{X })$ is the posterior probability of $z_i$ given the UAV data $\mathbf{X }$. It is in general difficult, if not impossible, to directly calculate the posterior probability $\Pr (z_i = j|\mathbf{X })$. We propose to iteratively learn the posterior probability and corresponding probability distributions by using Bayesian mixture model and Markov chain Monte-Carlo.

A multi-modal Bayesian mixture model is used to represent the probability distributions of the intensities of pixels in the UAV image, with each component in the mixture model corresponding to one possible category. The probability density function (pdf) of the *i*-th pixel can be represented as

5 $$\begin{aligned} f(\varvec{x}_i| \{\varvec{\theta }_j\}_{j=1}^{k}, \varvec{q}) = \sum _{j = 1}^{k} q_j f(\varvec{x}_i| z_i = j, \varvec{\theta }_j), \end{aligned}$$

where $f(\varvec{x}_i| z_i = j, \varvec{\theta }_j)$ is the likelihood function of $\varvec{x}_i$ given that the *i*-th pixel is in the *j*-th category, and $\varvec{\theta }_j$ is the corresponding distribution parameters of the *j*-th category. In Bayesian inference, $\varvec{\theta }_j$ is assumed to be unknown and random, with a prior distribution $\pi (\varvec{\theta }_j)$.

The multivariate Gaussian mixture model (GMM) is adopted in this paper, where the likelihood function is assumed to follow a Gaussian distribution with mean vector $\varvec{\mu }_j \in \mathcal{R}^{p \times 1}$ and covariance matrix ${\varvec{\Phi }}_j^{-1} \in \mathcal{R}^{p \times p}$ as

6 $$\begin{aligned} \varvec{x}_i|(z_i = j, \varvec{\theta }_j)\sim \mathcal{N}_p(\varvec{\mu }_j, {\varvec{\Phi }}_j), \end{aligned}$$

where the inverse of the covariance matrix, ${\varvec{\Phi }}_j$ is the precision matrix. Using precision matrix instead of the covariance matrix can reduce the number of matrix inversions in the learning process. The corresponding distribution parameters are thus $\varvec{\theta }_j = \{\varvec{\mu }_j, {\varvec{\Phi }}_j\}$. Under the Bayesian setting, the mean vector ${\varvec{\mu }}_j$ and precision matrix ${\varvec{\Phi }}_j$ are unknown and random.

The Bayesian posterior probability can then be calculated as

7 $$\begin{aligned} \Pr (z_i = j|\mathbf{X }) = \frac{\int _{\varvec{\theta }_j}\!\!\! \int _{q_j} q_j f(\mathbf{X }|z_i = j, \varvec{\theta }_j)\pi (\varvec{\theta }_j) \pi (q_j) d\varvec{\theta }_j dq_j}{f(\varvec{X})} \end{aligned}$$

The calculation of the posterior probability requires multi-level integration with respect to the multi-dimensional parameter $\varvec{\theta }_j$ and $q_j$, which are usually difficult to carry out either analytically or numerically. We propose to solve this problem by employing unsupervised Bayesian learning with Gibbs sampling [19, 20], and details are given in the next section.

### Unsupervised Bayesian learning with Gibbs sampling

In this section, an unsupervised Bayesian learning method with the Gaussian mixture model (GMM) is used to classify the pixels in the UAV images into one of several categories, such as panicles, leaves, dirt, water, etc. The classification is performed by analyzing and identifying the statistical properties of the pixels belonging to different categories, without the need of a training phase.

As in the problem formulated in (4) and (7), the classification requires the knowledge of the posteriori probability. MCMC with Gibbs sampling can obtain a numerical approximation of $\text {Pr}(z_i = j|\varvec{x}_i)$ by iteratively taking samples from the joint distribution

$$\begin{aligned} f(\varvec{z},\{\varvec{\theta }_j\}_{j = 1}^{k},\varvec{q}|\varvec{X}). \end{aligned}$$

For a given $\varvec{X}$, if *T* samples are drawn from the joint distribution and, and the samples are denoted as $\{\varvec{z}^{(\text {itr})}\}^{T}_{\text {itr}= 1}, \{\varvec{\theta }^{(\text {itr})}_j\}^{T}_{\text {itr}= 1}$ for $j = 1,2,\ldots ,k$ and $\{\varvec{q}^{(\text {itr})}\}^{T}_{\text {itr}= 1}$. Based on the law of large numbers, as $T \rightarrow \infty ,$

8 $$\begin{aligned} \text {Pr}(z_i = j|\varvec{X}) = \lim _{T \rightarrow \infty } \frac{1}{T} \sum _{itr = 1}^{T} \mathcal {I}(z_{i}^{(\text {itr})} = j), \end{aligned}$$

where $\mathcal {I}(\mathcal {E})$ is an indicator function defined as $\mathcal {I}(\mathcal {E}) = 1$ if $\mathcal {E}$ is true and 0 otherwise. The basic idea of MCMC with Gibbs sampling is to iteratively take samples based on the posterior distributions of different variables conditioned on previously taken samples.

#### Initialization

In order to start the iterative sampling process, the values of the unknown variables and parameters need to be initialized. The values obtained using results from *k*-means clustering [22, 23] are used as initial values. With the *k*-means algorithm, the pixels $\{\varvec{x}_i\}_{i = 1}^{n}$ are classified into *k* categories. Consider the set of pixels that correspond to the *j*-th category as $\mathcal {S}_{j}^{(0)} = \{i : z_i = j \}$ with cardinality $n_{j}^{(0)} = |\mathcal {S}_{j}^{(0)}|$. The vector $\varvec{z}^{(0)}$ is initialized by assigning $z_i^{(0)} = j$ if $i \in \mathcal {S}_{j}^{(0)}$. Then the vector $\varvec{q}^{(0)}$ is initialized as

9 $$\begin{aligned} q_j^{(0)} = \frac{n_{j}^{(0)}}{n}, \quad j = 1,2,\ldots ,k. \end{aligned}$$

Define a matrix $\varvec{X}_j = \{\varvec{x}_i\}_{i \in \mathcal {S}_{j}}$, which contains all pixels $\varvec{x}_i$ labeled as $z_i = j$.

The unknown parameters $\varvec{\theta }_j$ can then be estimated from $\varvec{X}_j$ by using maximum likelihood estimation. Under GMM, the unknown parameters are $\varvec{\theta }_j = \{\varvec{\mu }_j, {\varvec{\Phi }}_j\}$, and they can be initialized as

10 $$\varvec{\mu }_j^{(0)} = \frac{1}{n_j^{(0)}}\sum _{i \in \mathcal {S}_j^{(0)}} \varvec{x}_i$$

11 $${\varvec{\Phi }}_j^{(0)} = \left( \frac{1}{n_j^{(0)}}\sum _{i \in \mathcal {S}_j^{(0)}}(\varvec{x}_i - \varvec{\mu }_j)(\varvec{x}_i - \varvec{\mu }_j)^T \right) ^{-1}$$

#### Gibbs sampling with GMM

Gibbs sampling is used to iteratively take samples from the joint distribution $f(\varvec{z},\{\varvec{\theta }_j\}_{j = 1}^{k},\varvec{q}|\varvec{X})$. The results are then used to estimate the posteriori probability as in (8).

With GMM, the unknown model parameters are $\varvec{\theta }_j = \{\varvec{\mu }_j, {\varvec{\Phi }}_j\}$ for $j = 1,2, \ldots ,k$. Under the Bayesian setting, both ${\varvec{\mu }}_j$ and ${\varvec{\Phi }}_j$ are assumed to be unknown and random, and their values will be learned from the data. The prior for the mean vector $\varvec{\mu }_j$ is assumed to be Gaussian distributed with mean vector ${\varvec{\tau }}_j$ and precision matrix ${\varvec{\Omega }}_j$ as

12 $$\begin{aligned} \varvec{\mu }_j \sim \mathcal {N}_p({{{\varvec{\tau }}_j},{{\varvec{\Omega }}_j}}). \end{aligned}$$

The parameters ${\varvec{\tau }}_j$ and ${\varvec{\Omega }}_j$ will be iteratively updated during the Gibbs sampling process.

The non-informative prior [26] for precision matrix ${\varvec{\Phi }}_j$ is taken as

13 $$\begin{aligned} \pi ({{\varvec{\Phi }}_j}) \propto |{{\varvec{\Phi }}_j}|^{(p+1)/2} \end{aligned}$$

During the iterative Gibbs sampling process, the samples of different variables at each step are drawn based on their respective posterior distributions, conditional on current states of all other variables. Thus the implementation of Gibbs sampling requires the knowledge of full conditional posterior distribution of all parameters of interests which include $\varvec{z}, \varvec{q}, \{\varvec{\mu }_j\}_{j=1}^{k},$ and $\{{\varvec{\Phi }}_j\}_{j=1}^{k}$. The full conditional posterior distributions of all parameters are given as follows. Detailed derivations of (14)–(17) are given in Appendix.

*Posterior distribution of*$\varvec{q}$ Let $n_j$ denote the number of pixels belonging to the *j*-th category, then

14 $$\begin{aligned} \varvec{q | z} \sim \text {Dir}(\varvec{\alpha }+\varvec{n}). \end{aligned}$$

where $$\varvec{n} = [n_1, n_2, \ldots , n_k]^T$$.

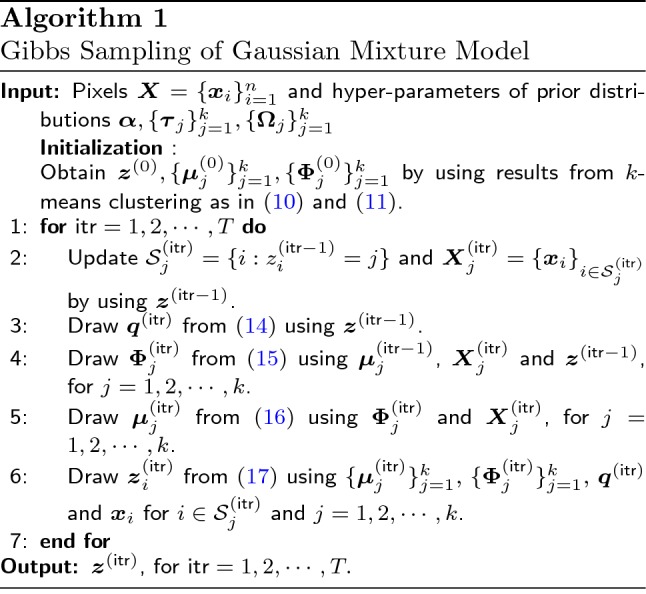



*Posterior distribution of*${\varvec{\Phi }}_j$*(non-informative Prior)*

15 $$\begin{aligned} {\varvec{\Phi }}_j|(\varvec{\mu }_j,\varvec{X}_j) \sim \mathcal {W}_p(\varvec{S}_j^{-1},n_j), \end{aligned}$$

where $\varvec{S}_j = \sum _{i \in \mathcal {S}_j} (\varvec{x}_i-\varvec{\mu }_j)(\varvec{x}_i-\varvec{\mu }_j)^T$, $\mathcal{S}_j = \{i: z_i = j \}$, and $\mathcal {W}_p(\varvec{S}_{j}^{-1},n_j)$ is Wishart distribution with $n_j$ degrees-of-freedom.

*Posterior distribution of*$\varvec{\mu }_j$

16 $$\begin{aligned} \varvec{\mu }_j|({\varvec{\Phi }}_j,\varvec{X}_j) \sim \mathcal {N}_p(\varvec{\tau }_j^*,\varvec{\Omega }_j^*), \end{aligned}$$

where $\varvec{\Omega }_j^*= n_j{\varvec{\Phi }}_j + \varvec{\Omega }_j$ and $\varvec{\tau }_j^* = \varvec{\Omega }_j^{*-1} (n_j{\varvec{\Phi }}_j \bar{\varvec{x}}_j + \varvec{\Omega }_j \varvec{\tau }_j).$ Here, $\bar{\varvec{x}}_j = \frac{1}{n_j} \sum _{i \in \mathcal {S}_j} \varvec{x}_i.$

*Posterior distribution of*$z_j$

17 $$\begin{aligned} \Pr (z_i = j |\varvec{x}_i,\{\varvec{\theta }_j\}_{j = 1}^{k},\varvec{q}) = \frac{q_j f(\varvec{x}_i |z_i = j, \varvec{\mu }_j, {\varvec{\Phi }}_j)}{\sum _{v = 1}^{k} q_v f(\varvec{x}_i |z_i = v, \varvec{\mu }_v, {\varvec{\Phi }}_v)}, \end{aligned}$$

where $${\varvec{x_i}|(z_i = j, \varvec{\mu }_j, {\varvec{\Phi }}_j) \sim } \mathcal {N}_p(\varvec{\mu }_j, {\varvec{\Phi }}_j)$$.

The Gibbs sampling algorithm with GMM is summarized in Algorithm 1.

As the number of iterations grows large, the samples drawn through this process converge to their joint distributions. With such a process, the values of all model parameters are learned from the data without the need of a training process. The output of the Gibbs sampling algorithm is then used to evaluate the posterior probability $\text {Pr}(z_i = j|\mathbf{X })$ to obtain an estimate of $\hat{z_i}$.

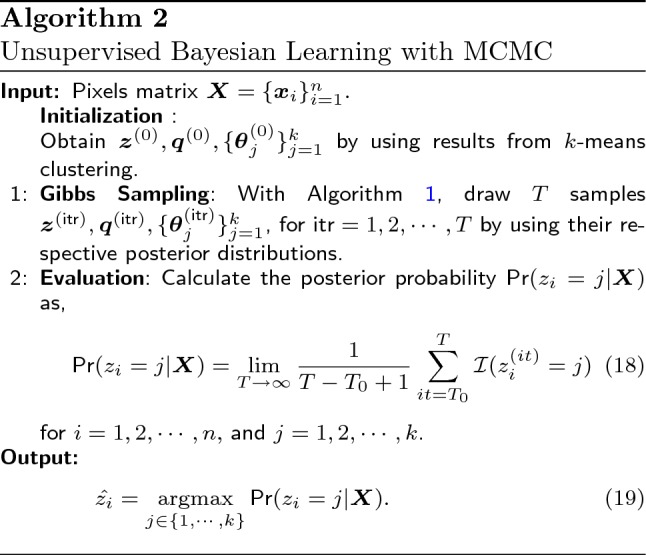

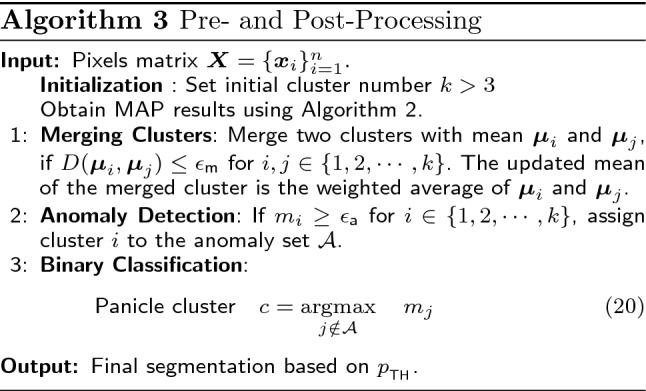





#### Unsupervised Bayesian learning with MCMC

The outline of the unsupervised Bayesian learning algorithm with MCMC is summarized in Algorithm 2 with the initial values of $\varvec{z}^{(0)}, \varvec{q}^{(0)}$ and $\varvec{\theta }_j^{(0)}.$ In Gibbs sampling, the generated samples at the beginning of the sampling process usually do not represent the actual joint distribution. Therefore, first $T_0 -1$ samples are usually discarded during the evaluation process as shown in (18).

It is important to highlight that since there is not a natural ordering between mixture components, it is necessary to label them for their posterior identification. In this proposed algorithm, the label of the components are ordered according to total mean across all channels i.e. under this assumption the panicle segments have the highest total mean across all channels.

#### Pre- and post-processing

In order to account for diverse illumination conditions and the presence of anomalous objects, the classification is started with a relatively large number of clusters $k > 3$. After classification with $k > 3$, two additional steps are performed to merge close clusters and to detect and remove anomalies. Details are given in Algorithm 3.

In step 1 (‘Merging Clusters’), two clusters with mean ${\varvec{\mu }}_i = [\mu _{i, r}, \mu _{i, g}, \mu _{i, b}]^T$ and ${\varvec{\mu }}_j = [\mu _{j, r}, \mu _{j, g}, \mu _{j, b}]^T$ are merged into a single cluster if the Euclidean distance between the two means, $D({\varvec{\mu }}_i,{\varvec{\mu }}_j)=\Vert {\varvec{\mu }}_i - {\varvec{\mu }}_j\Vert$, is less than a predefined threshold $\epsilon _\text {m}$, where $\Vert \mathbf{a }\Vert$ is the $\ell _2$-norm of the vector $\mathbf{a }$. In this paper $\epsilon _\text {m}= 0.1$ has been used. The mean of the merged cluster is calculated as the weighted average of ${\varvec{\mu }}_i$ and ${\varvec{\mu }}_j$. This step deals with different illumination conditions assuming a class gets classified in two or more different classes because of illumination.

In step 2 (‘Anomaly Detection’), cluster *i* is classified as anomaly if the total mean across all three channels, $m_i = \sum _{j \in \{r,g,b\}} \mu _{i,j}$, is above a predefined threshold $\ge \epsilon _\text {a}$. In this paper, $\epsilon _\text {a}= 0.9$ has been used for anomaly detection.

## Data Availability

The dataset analyzed during the current study are available at https://wuj.hosted.uark.edu/research/datasets/panicle/UBLRPSUI.zip. Also, the MATLAB code and related materials can be downloaded from https://github.com/i2pt/UBLRPSUI.

## Appendix

### Derivations of full conditional posteriori distributions

Detailed derivations of the full conditional posterior distributions in (14)–(17) are given in this appendix.

#### Posterior distribution of $\varvec{q}$

Based on the assumption of Dirichlet prior, using Bayes’ rule, the posterior distribution of $\varvec{q}$ can be written as

$$\begin{aligned} f(\varvec{q}|\varvec{z})&= \frac{f(\varvec{z}|\varvec{q}) \cdot \pi (\varvec{q})}{f(\varvec{z})} \propto \prod _{j=1}^{k} q_{j}^{n_j} \prod _{j=1}^{k} q_{j}^{\alpha _j -1} \\&\propto \prod _{j=1}^{k} q_{j}^{\alpha _j + n_j -1}, \end{aligned}$$

where $n_j$ is the number of pixels labeled in the *j*-th category. As can be seen from the equation, the posterior distribution of $\varvec{q}$ also follows Dirichlet distribution as

$$\begin{aligned} \pi (\varvec{q | z})&= \text {Dir}([\alpha _1+n_1, \alpha _2+n_2, \ldots , \alpha _k+n_k]^T) \\&= \text {Dir}(\varvec{\alpha }+\varvec{n}). \end{aligned}$$

#### Posterior distribution of ${\varvec{\Phi }}_j$ (non-informative prior)

For all the following proofs it is assumed that prior distributions $\pi (\varvec{\mu }_j)$ and $\pi ({\varvec{\Phi }}_j)$ are independent. Hence, $\pi (\varvec{\mu }_j,{\varvec{\Phi }}_j) = \pi (\varvec{\mu }_j)\pi ({\varvec{\Phi }}_j)$. Assume $\pi ({\varvec{\Phi }}_j) \propto |{\varvec{\Phi }}_j|^{\frac{p+1}{2}}$, then

$$\begin{gathered} f\left( {\left. {\Phi _{j} } \right|\mu _{j} ,X_{j} } \right) \propto f\left( {\left. {X_{j} } \right|\mu _{j} ,\Phi _{j} } \right)\pi \left( {\Phi _{j} } \right) \hfill \\ \propto \left| {\Phi _{j} } \right|^{{\frac{{{\text{n}}_{j} }}{2}}} \exp \left\{ { - \frac{1}{2}\sum\limits_{{i \in S_{j} }} {\left( {x_{i} - \mu _{j} } \right)^{{\text{T}}} \Phi _{j} \left( {x_{i} - \mu _{j} } \right)} } \right\} \hfill \\ \times \left| {\Phi _{j} } \right|^{{\frac{{{\text{n + 1}}}}{2}}} \hfill \\ \propto \left| {\Phi _{j} } \right|^{{\frac{{{\text{n}}_{j} + p + 1}}{2}}} \exp \left\{ { - \frac{1}{2}{\text{tr}}\left( {S_{j} \Phi _{j} } \right)} \right\} \hfill \\ \end{gathered}$$

where $$\varvec{S}_j = \sum _{i \in \mathcal {S}_j} (\varvec{x}_i-\varvec{\mu }_j) (\varvec{x}_i-\varvec{\mu }_j)^T$$. Thus

$$\begin{aligned} {\varvec{\Phi }}_j|(\varvec{\mu _j},\varvec{X}_j) \sim \mathcal {W}_p(\varvec{S}_j^{-1},n_j), \end{aligned}$$

a *p*-variate Wishart distribution with $n_j$ degrees-of-freedom.

#### Posterior distribution of $\varvec{\mu }_j$

The posterior distribution of ${\varvec{\mu }}_j$ can be calculated as

$$\begin{gathered} f\left( {\left. {\mu _{j} } \right|\Phi _{j} ,X_{j} } \right) \propto f\left( {\left. {X_{j} } \right|\mu _{j} ,\Phi _{j} } \right)\pi \left( {\mu _{j} } \right) \hfill \\ \propto \exp \left\{ { - \frac{1}{2}\sum\limits_{{i \in S_{j} }} {\left( {x_{i} - \mu _{j} } \right)^{{\text{T}}} \Phi _{j} \left( {x_{i} - \mu _{j} } \right)} } \right\} \hfill \\ \times \exp \left\{ { - \frac{1}{2}\left( {\mu _{j} - \tau _{j} } \right)^{{\text{T}}} \Omega _{j} \left( {\mu _{j} - \tau _{j} } \right)} \right\} \hfill \\ \propto \exp \left\{ { - \frac{1}{2}\left( {\mu _{j}^{{\text{T}}} \Omega _{j}^{*} \mu _{j} - 2\mu _{j}^{{\text{T}}} \user2{A}} \right)^{{\text{T}}} } \right\}, \hfill \\ \end{gathered}$$

where $\varvec{\Omega }_j^*= n_j{\varvec{\Phi }}_j + \varvec{\Omega }_j$ and $\varvec{A} = n_j{\varvec{\Phi }}_j \bar{\varvec{x}}_j + \varvec{\Omega }_j \varvec{\tau }_j$. Hence,

$$\begin{aligned} \varvec{\mu }_j|{\varvec{\Phi }}_j,\varvec{X}_j \sim \mathcal {N}_p(\varvec{\tau }_j^*,\varvec{\Omega }_j^*) \end{aligned}$$

where $$\varvec{\tau }_j^* = \varvec{\Omega }_j^{*-1} (n_j{\varvec{\Phi }}_j \bar{\varvec{x}}_j + \varvec{\Omega }_j \varvec{\tau }_j).$$

#### Posterior distribution of $\varvec{z}_j$

The posteriori distribution in (17) can be directly obtained by applying the Bayes’ rule.

## Abbreviations

UAV: Unmanned aerial vehicle
GMM: Gaussian mixture model
MCMC: Markov chain Monte Carlo
ANN: Artificial neural network
CNN: Convolutional neural network
SVM: Support vector machine
PCA: Principle component analysis
